# Minute Anatomy of Fatty Degeneration of the Liver

**Published:** 1842-08

**Authors:** 


### Selections from American and Foreign Journals.

---

*Minute Anatomy of Fatty Degeneration of the Liver.*—The author observes, that in order to make the subsequent description intelligible, he will premise a few words on the minute structure of the lobules of the liver.

Mr. Kiernan has well described the *vascular element* of these minute representatives of the organ. It consists of a capillary plexus intervening between the portal and hepatic veins. The diameter of the capillaries in this plexus is very large, being nearly twice that of a blood globule; while the diameter of the capillaries in most other textures is the same as that of the blood-globule, and in some (as the muscular) even less, so that the blood-globules only pass along by undergoing elongation. This large size of the capillaries of the liver, probably, has reference to the deficiency of propelling power in the portal circulation. This *portal hepatic plexus* may be termed *solid*, as it is extended in all directions, and presents areolæ of nearly the same dimensions in whatever plane it is cut. These areolæ are in general not larger than the diameter of the vessels which form them, so that a well-injected specimen might appear to be composed of little else than vessels.

In the interstices of this capillary plexus lies the *secreting portion of the bile-ducts.* If a thin section of an uninjected lobule be examined with a sufficient magnifying power, it is seen to be almost entirely made up of small, irregular, angular particles, each containing a circular or oval nucleus, within which is a minute point or two, the nucleolus. These particles have a determinate outline, are of some thickness, and possess a fine granular aspect. They also contain (which is very remarkable) one, two, or more globules of fatty matter, irregularly placed, and of somewhat variable bulk.

The microscope at once reveals the seat of the *fatty deposite in the diseased state of the organ.* Instead of containing a few minute scattered globules, *the nucleated particles are gorged with large masses of it*, which greatly augment their bulk, and more or less obscure their nuclei.

This simple description developes the whole anatomical condition of the disease, as well as explains its rougher characters, the bulk, the color, and the freedom of the circulation. The particles, lying in the interstices of the capillary plexus, enlarge slowly and equably, in such a manner as to exert no injurious pressure on the vessels, while their new contents impart that peculiar hue which characterizes the disease. It also throws no little light on the nature and source of the disease. It seems to show that the fat is an *increase of a normal constituent*, and not a formation altogether unnatural in kind; thus distinguishing it from the fatty degenerations of other tissues, where fat is deposited in situations from which it is naturally absent. It likewise indicates an increased activity in the secreting action of the liver, for a considerable period before death, though why the *accumulation* of fat should occur within the nucleated particles does not so clearly appear. To explain that fully, will require a more complete knowledge than we yet possess of the chemical affinities at play within these small laboratories of nature.—Mr. Bowman, *Lancet, Jan.* 1842.

